# Safety of Botulinum Toxin A in Children and Adolescents with Cerebral Palsy in a Pragmatic Setting

**DOI:** 10.3390/toxins5030524

**Published:** 2013-03-12

**Authors:** Antigone S. Papavasiliou, Irene Nikaina, Katerina Foska, Panagiotis Bouros, George Mitsou, Constantine Filiopoulos

**Affiliations:** 1 Department of Neurology, Pendeli Children’s Hospital, 8 Hippokrates street, Palaia Penteli 15236, Athens, Greece; E-Mails: enikaina@gmail.com (I.N.); dkfoska@gmail.com (K.F.); itheka@otenet.gr (P.B.); gmitsou@hotmail.com (G.M.); 2 Department of Orthopedics, Pendeli Children’s Hospital, 8 Hippokrates street, Palaia Penteli 15236, Athens, Greece; E-Mail: konfilips@gmail.com

**Keywords:** cerebral palsy, spasticity, treatment, botulinum toxin A, onabotulinumtoxin A, abobotulinumtoxin A, adverse events, safety

## Abstract

This retrospective study aimed to examine the safety of botulinum toxin A (BoNT-A) treatment in a paediatric multidisciplinary cerebral palsy clinic. In a sample of 454 patients who had 1515 BoNT-A sessions, data on adverse events were available in 356 patients and 1382 sessions; 51 non-fatal adverse events were reported (3.3% of the total injections number, 8.7% of the patients). On five occasions, the adverse reactions observed in GMFCS V children were attributed to the sedation used (rectal midazolam plus pethidine; buccal midazolam) and resulted in prolongation of hospitalization. Of the reactions attributed to the toxin, 23 involved an excessive reduction of the muscle tone either of the injected limb(s) or generalized; others included local pain, restlessness, lethargy with pallor, disturbance in swallowing and speech production, seizures, strabismus, excessive sweating, constipation, vomiting, a flu-like syndrome and emerging hypertonus in adjacent muscles. Their incidence was associated with GMFCS level and with the presence of epilepsy (Odds ratio (OR) = 2.74 − *p* = 0.016 and OR = 2.35 − *p* = 0.046, respectively) but not with BoNT-A dose (either total or per kilogram). In conclusion, treatment with BoNT-A was safe; adverse reactions were mostly mild even for severely affected patients. Their appearance did not necessitate major changes in our practice.

## 1. Introduction

Most children identified with Cerebral Palsy (CP) present with spasticity (77%), with bilateral spastic CP being more common than unilateral spastic CP [1]. The need for treating spasticity is crucial in the developing child, because it may interfere with muscle growth that could produce deformity, as well as with daily activity [2,3]. Physiotherapy, oral medications, botulinum toxin, phenol, selective posterior rhizotomy, intrathecal baclofen and orthopaedic surgery are used towards this end; among the surgical or conventional intervention strategies used in the management of spasticity, botulinum toxin type A (BoNT-A) has acquired an important role [4,5]. Botulinum toxin type A used as a treatment option in CP is considered to be safe, especially in ambulatory children [6,7]. Even though the experience of multiple groups has not produced serious adverse event rates that necessitated major changes in clinical practice the concern for adverse effects has been universal [8]. Worries about the safety of BoNT-A injections emerged particularly after 2005, when treatment with the toxin was associated with 28 deaths [9]. Two years later Howell *et al.* reported deterioration in respiratory and oromotor function in a boy with severe cerebral palsy following repeated injections of BoNT-A [10] and they advised caution when this drug is used to treat hypertonicity in children with severe cerebral palsy and pseudobulbar palsy. As a result of those reports, in 2008, a black box warning appeared on labels of botulinum toxin that require warning of the risk of the spread of botulinum toxin beyond the injection site with the associated risks of dysphagia, aspiration and/or pneumonia and death [11]. This warning subsequently produced some changes in practice for the more severely affected patients and more specifically, for patients who according to the Gross Motor Function Classification System for Cerebral Palsy (GMFCS) belong to levels IV and V. For example, in 2010, following the death of child with CP, GMFCS level V, some physicians decided to stop treating with BoNT-A injections children at GMFCS level V (except for some indications such as pain relief) and set an upper dose limit of 18 IU⁄kg for children at GMFCS level IV [12]. More recently, O’Flaherty *et al.* conducted a prospective pre-post cohort study of children undergoing this treatment in a single children’s rehabilitation department over a 16-month period and determined that in the month following injections, an adverse event occurred in 23% of injection episodes, 2% of these were potentially serious (lower respiratory tract infections/ aspiration, worsening dysphagia, or generalized weakness), but this rate was slightly lower than, or no different from, the rate of these events in the month before the injections [13].

Concerning the type of the adverse events, a great number (more than 30) of different events were reported. BoNT-A use was associated with respiratory tract infections, bronchitis, pharyngitis, asthma, muscle weakness, urinary incontinence, falls, seizures, fever and unspecified pain [12,14]. In their study, Olesh *et al.* tested the safety of three repeated botulinum toxin injections in the upper limb of 22 children with CP [15]. Only three patients (13.6%) experienced mild side effects (e.g., local or more extensive weakness, and a maculopapular rash). Russo *et al.* described excessive limb weakness, a flu-like syndrome, episodes of headache, vomiting and cough in children after BoNT-A injection in their upper limbs [16]. Adverse events attributed to systemic spread of the toxin are uncommon and include flulike symptoms, generalized weakness, dysphagia, and subsequent aspiration caused by diminished airway protection [13]. In a review including 20 RCTs and 882 participants, death was reported in two cases (in different studies) and it was observed in the two patients with the longest follow up time. None of these was found to have a causal relation to the toxin [14]. 

The objectives of this report were to examine the safety of BoNT-A two preparations (Onabotulinumtoxin A^®^ & Abobotulinumtoxin A^®^) for this population and particularly for GMFCS IV, V levels.

## 2. Patients and Methods

### 2.1. Setting

This is a comprehensive multidisciplinary clinic in one of the three Children’s Hospitals serving the Athens Metropolitan area (population 4.5 million) and accepting referrals from the entire country. This pragmatic study was conducted in a National Health System department; patient data were recorded by the clinicians who provided care, according to a protocol that was prepared when the spasticity management service was established. The protocol was at times modified according to emerging practice guidelines, however, procedures such as patient assessment prior to the injections by child neurologist (ASP), pediatric orthopedic surgeon (CF) and NDT therapist (PB) as well as scheduled re-evaluations remained basically steady over the 13 years that this clinic has provided patient services.

### 2.2. Patients

Consecutive children and adolescents with a diagnosis of Unilateral or Bilateral Spastc Cerebral Palsy who received BoNT-A injections for spasticity management between 1999 and 2012 were included in this study.

Inclusion Criteria: Diagnosis of cerebral palsy; Age < 18 years; utilization of BoNT-A; ongoing physical and/or occupational therapy for a minimum of two 45-minute sessions per week, with the therapy regimen left to the discretion of the treating therapists; signed informed consent, obtained from capable participants and their guardians.

Exclusion Criteria: Dystonia not related to cerebral palsy; Spasticity resulting from traumatic brain or spinal cord Injury and stroke in older children, familial spastic paraparesis and other conditions presenting with spasticity but not belonging to the group of disorders characterized as CP; Age > 18 years. 

### 2.3. Procedures

Botulinum toxin injections were recommended for specific indications following neurologic examination, GMFCS classificationand several functional tests and outcome measurements, according to existing treatment guidelines [17,18]. GMFCS is a reliable and valid system that has been widely utilized for objective classification of the patterns of motor disability in children with cerebral palsy [19]. GMFCS classifies CP into 5 levels, from level I (the most independent motor function) to level V (the most restricted voluntary control of movement and ability to maintain antigravity head and trunk postures). In this study, the GMFCS-Greek version was used in order to classify our patients; this tool has been used by our group for many years and its reliability has been demonstrated [20]. A pamphlet in Greek language has been produced and utilized for hands on training of our trainees and therapists. 

The protocol called for multi-level botulinum toxin A injections in the upper and/or lower limbs, repeated as needed at greater than 4 months intervals and performed during a short hospital admission (from 8 a.m. to 3 p.m). The drugs utilized were Onabotulinumtoxin A (Botox^®^, Allergan Inc, Westport, County Mayo, Ireland) and Abobotulinumtoxin A (Dysport^®^, Ipsen Ltd, Wrexham, UK). The doses utilized from 1999 to 2005 were for Onabotulinumtoxin A, 12 to 20 IU/kg/session or maximum total 400 IU/session and for Abobotulinumtoxin A, 30 to 50 IU/kg/session or maximum total 2000 IU/session. Based on our clinical experience and reports from the literature, after 2006, Onabotulinumtoxin A doses were modified to maximum 25 IU/kg/session or up to total 500 units per session and for Abobotulinumtoxin A to 30 IU/kg/session or maximum total 1500 IU/kg/session [17,18].

We have injected the great majority of our patients under conscious sedation. BoNT-A was administered after local anesthesia with EMLA cream and from 1999 to 2004 after rectal administration of midazolam 0.2–0.3 mg/kg and pethidine 1 mg/kg. Starting in 2005, buccal midazolam at a dose of 0.3 mg/kg/dose with maximum dose 1 mg/session was utilized. General anaesthesia was used infrequently and only after injection of the psoas muscle was deemed absolutely necessary for the management of our patients; this limitation was the result of limited coverage of our clinic by the department of anaesthesiology. 

Injection technique: Starting in 1999 manual muscle testing and electrical stimulation were used in order to localize the muscles that we had decided to inject. Starting in 2009 muscle ultrasound technique has been utilized. 

#### 2.3.1. Patient Monitoring

In the process of obtaining informed consent potential benefits and possible adverse events were discussed with patients and caregivers. Particular explanations were given for patients with known risk factors and parents were instructed on how to identify signs and symptoms of adverse events such as, difficulty swallowing, talking, breathing, or generalized muscle weakness; the need to seek immediate medical attention was stressed.

#### 2.3.2. Adverse Events Reporting

Clinical response to treatment was assessed at 3, 6 and 12 months post-treatment and untoward events following the BoNT-A injections and their timing were recorded; continuation or discontinuation of BoNT-A injections was recommended based on efficacy and safety of previous treatments. There were structured questions based on the listed side effects provided by the pharmaceutical companies as well as the existing literature such as, excessive hypotonia, generalized and focal, chewing and swallowing disturbances, aspiration, pneumonia, constipation and enuresis [11,12,13,14]. The parents and caregivers were also requested to report whatever untoward event they observed following the injection whether or not they believed that this was related to this treatment. All untoward events were rated as mild, moderate or severe. The parents of all patients who did not have an evaluation visit after their final injection were contacted by phone and asked for the possible occurrence of the adverse events above mentioned as well as their temporal relationship to the injections. 

### 2.4. Statistical Analysis

Continuous data were tested for normality using the Kolmogorov Smirnov test. Normally distributed continuous variables were expressed as mean values (±standard deviation) whereas non-normal data were expressed as median values (interquantile range). Categorical data were presented as frequencies and percentages. Comparisons between continuous variables were based on *t*-test if variables were normally distributed and on Mann-Whitney *U* test when this was not the case. Frequencies of categorical variables were compared with chi-square or Fisher’s exact test. 

In order to discover the factors predicting the appearance of an adverse reaction, we performed a logistic regression analysis. Factors tested in the univariate analysis were sex (male *vs*. female), duration of gestation (preterm *vs*. full term), localization of the involvement (bilateral *vs.* unilateral), medication used (Onabotulinumtoxin A *vs*. Abobotulinumtoxin A), severity of the impairment (GMFCS: I–III *vs*. GMFCS: IV–V), age at treatment initiation (>4 years old *vs*. ≤4 years old) and the presence or not of an epileptic disorder. All variables that proved to be significant or with a *p*-value ≤ 0.200 at the univariate analysis were inserted in the multivariate analysis. All analyses were performed with STATA 9.2 statistical package (StataCorp LP; College Station; TX, USA).

## 3. Results

Four hundred and fifty four patients with CP out of the total of 506 were treated with BoNT-A injections in our department (89.7%), fulfilled inclusion criteria for this report. The patients that we treated with BoNT-A but were excluded from this analysis belonged to three groups: most were treated for spasticity due to traumatic brain Injury and stroke that happened in late childhood, familial spastic paraparesis and other conditions presenting with spasticity but not belonging to the CP group, many were treated for dystonia related to neurometabic or neurodegenerative disorders (for example, glutaric aciduria, Wilson’s disease *etc.*) and some had CP but they were given BoNT-A injections at an age older than 18 years for the first time. Of the 454 patients, 356 had been available for an interview on adverse events. The remaining 98 either were lost to follow-up or, although contacted, could not be scheduled for an appointment after their most recent injection. They were 162 females and 195 males, with a mean age of 5.1 years (SD 3.4 years, range 1.5–18 years). Seventy nine children had unilateral spastic cerebral palsy and 252 had bilateral spastic cerebral palsy. As for the severity of the impairment, the majority of the studied patients (65.45%) were categorized as GMFCS levels I, II, III and 34.55% belonged to GMFCS levels IV and V. Severity of impairment was similar in the two sex groups (*p* = 0.459) and in the two age groups (<4 years and ≥4 years, *p* = 0.458). However, children with bilateral involvement, as a group, were more severely affected, compared to those with unilateral involvement (only 8% of children with unilateral involvement had a GMFCS IV–V, whereas this percentage reached 44.2% in children with bilateral involvement) (*p* < 0.001). Follow up time ranged between 3 months and 11 years 9 months. 

### 3.1. Botulinum Toxin Injection

One thousand three hundred and eighty two sessions for BoNT-A injections were performed in patients included the analysis of adverse events; lower limbs were injected in 92.2% of the sessions and the upper limbs in 31.9%. The median injections number per patient was 3 (range 1–20), with only 13 patients having more than 10 injection sessions. The preparation used for the injections was Onabotulinumtoxin A in 70.4% and Abobotulinumtoxin A in 29.6% of the patients. Doses of BoNT-A, calculated per kilo weight, had at the first visit median value of 13.1 IU/kg (IQR: 10–16.3 IU/kg, max: 20.9 IU/kg) for Onabotulinumtoxin A and 26.7 IU/kg (IQR: 20–34.3 IU/kg, max: 59.1IU/kg) for Abobotulinumtoxin A. The dosages used before and after the release of the first European Concensus Report in 2006 [23] were similar for Onabotulinumtoxin A dose per kilo was 15 IU/kg (IR: 11.2–18.6 IU/kg, max: 26.3 IU/kg) before 2006 *vs*. 14.2 IU/kg (IR: 10–18.2 IU/kg, max: 29.7 IU/kg) after 2006, (*p* = 0.118) but were different for Abobotulinumtoxin A 35.7 IU/kg (IR: 27.7–46.7 IU/kg, max: 59.5 IU/kg) before 2006 *vs*. 27.5 IU/kg (IR: 21.6–30 IU/kg, max: 43.5 IU/kg) after 2006, (*p* < 0.001). Only few patients (*n* = 4) received Abobotulinumtoxin A exceeding 40 IU/kg after 2006. Dose conversion ratios of the two regimens used (Onabotulinumtoxin A: Abobotulinumtoxin A) had also changed before and after the application of the guidelines from 1:2.4 before to 1:1.9 after 2006. 

### 3.2. Adverse Events

Over 13 years and 1382 injection sessions we had five serious adverse reactions that were attributed to the two regimens of conscious sedation that we have adopted in this clinic; these necessitated prolongation of the hospitalization of the patients. Three incidents were observed during the period of rectal administration of midazolam and pethidine and two during the period of buccal midazolam. These were observed in five GMFCS V children and were characterized by deep sedation, swallow breathing and decreased oxygen saturation of the hemoglobin. On two occasions the sedation resolved within 3–4 h; on the other three, full recovery to their previous state was accomplished by the following morning. In the first two patients, hospitalization was prolonged for 12 h and for the remaining three, overnight hospitalization was deemed necessary. One child remained in the ICU for observation overnight due to persistently low SaO2. This was a 10 year-old quadriplegic girl with history of frequent aspiration events for whom we had recommended gastrostomy placement that had not been accepted by the family; she fully recovered within 12. None of these patients developed symptoms of botulism in the ensuing days and this is why these events were attributed to the drugs used for sedation and not to BoNT-A. Besides the reactions mentioned above, 46 other adverse reactions were reported in a total of 1382 injections (3.3%) within one month after the injection session; these events involved 31 patients (8.7% of patients participating in the study).

The majority of these reactions (*n* = 23) involved a marked/excessive reduction of the muscle tone, both restricted to the limb injected and generalized. Its duration ranged between 1 day and 2 months. Table 1 displays the type of adverse events reported and their frequencies. In one patient the BoNT-A injection was reported to be followed by an increase in limb tone and in another dystonic features with right rotation of the trunk were described; neither of these events was verified by us because the patients were not brought to the hospital for examination. Local pain and restlessness were a concern in one patient and lethargy associated with pallor in three of them. More serious reactions were even less frequent. Disturbance in swallowing was reported in three patients, two in GMFCS levels IV and V and one in GMFCS level II; no aspiration or pneumonia were reported. Speech production disturbance was reported in two patients; in one of them this reportedly lasted for about one year. Other adverse events observed were seizures, strabismus, excessive sweating, constipation, vomiting and a flu-like syndrome. In none of these occasions, consultation, hospitalization or other action was felt to be necessary. No reaction leading to death was reported. Most reactions were observed after the first injection session and less frequently after the subsequent ones. No adverse reaction was reported after the fifth session. In the majority of the patients, adverse reactions were observed only once and reaction reappearance (always of the same type) was observed in five children. In these cases, the reaction was muscle tone disturbance. Patients with a more serious impairment had more adverse effects, as compared to those less handicapped (14.03% *vs*. 5.1%, chi2(1) = 7.94 *p* = 0.005). Total doses of the toxin injected did not correlate with the presence of an adverse event: for total doses for Onabotulinumtoxin A > 400 UI *vs*. ≤ 400 UI (*p*-value = 0.898) and for Abobotulinumtoxin A > 500 UI *vs*. ≤ 500 UI (*p* = 0.091). Doses per kilo of BoNT-A injected that were associated with the appearance of the reactions were: for Abobotulinumtoxin A median value = 37 mg/kg (range: 24.4–59.3 mg/kg) and for Onabotulinumtoxin A 15.8 mg/kg (range: 8.7–25.8 mg/kg). Very few children were injected with Abobotulinumtoxin A doses > 50 UI/kg before 2006 and 2 of them presented a reduction of the limb tone and generalized weakness respectively. After 2006, only four patients were injected with Abobotulinumtoxin A doses slightly above 40 UI/kg and none of them had an adverse event. To further explore the correlation between the dose per kilo of the regimen used, we categorized it as follows: for Onabotulinumtoxin A (0–4.99 UI/kg, 5–9.99 UI/kg, 10–14.99 UI/kg, 15–19.99 UI/kg, ≥20 UI/kg) and for Abobotulinumtoxin A (0–9.99 UI/kg, 10–19.99 UI/kg, 20–29.99 UI/kg, ≥30 UI/kg). Again, the dose per kilo did not influence the rate of adverse reactions (*p* = 0.656 − for Onabotulinumtoxin A and *p* = 0.249 − for Abobotulinumtoxin A).

**Table 1:** Frequency of retrospectively reported adverse reactions after botulinum toxin A (BoNT-A) injections in children with Cerebral Palsy (CP).

Type of adverse reaction	Number of reaction (%) *
Weakness (Generalized/Trunk/Limb)	6 (0.43%)/5 (0.36%)/12 (0.86%)
Other muscle tone disturbances (Hypertonia/Dystonia)	2 (0.14%)/1(0.07%)
Difficulty in swallowing	3 (0.22%)
Speech disturbances	2 (0.14%)
Seizures	1 (0.07%)
Constipation/vomiting/anorexia	1(0.07%)/1 (0.07%)/1(0.07%)
Pain	3 (0.22%)
Strabismus	1 (0.07%)
Excessive sweating	1 (0.07%)
Pallor	3 (0.22%)
Sleep disturbances	1 (0.07%)
Lethargy	5 (0.36%)
Sialorrhea	1 (0.07%)
Flu like syndrome	1 (0.07%)

* absolute number of adverse reactions (percentage of the total injection sessions e.g., *n* = 1382).

In order to reveal the factors associated with the presence of an adverse reaction we fitted a logistic regression model. According to the univariate analysis, significant predicting factors were: The GMFCS level and the presence of epilepsy (Table 2). On the other hand, the bilateral *vs*. unilateral involvement, the age at treatment onset, the date of first injection and the use of different medication (Onabotulinumtoxin A *vs*. Abobotulinumtoxin A) did not appear to influence the rate of adverse reactions.

**Table 2:** Univariate logistic regression analyses for the prediction of the appearance of an adverse reaction after BoNT-A injection.

Variables	OR	*p*-value	95% CI
Sex (male *vs*. female)	0.82	0.619	0.38–1.78
Birth (preterm *vs*. full term)	1.125	0.813	0.42–2.98
Bilateral vs unilateral involvement	1.16	0.752	0.45–2.98
GMFCS (IV–V *vs*. I–III)	3.04	0.007	1.36–6.80
Age (>4 years *vs*. ≤4 years)	1.35	0.455	0.61–2.97
Epilepsy	2.47	0.032	1.08–5.63
Onabotulinumtoxin A *vs*. Abobotulinumtoxin A	1.14	0.779	0.46–2.82
Year at treatment onset (before 2007 *vs*. after 2007)	0.75	0.484	0.33–1.69

Those two independent factors remained significant in the multivariate logistic regression model fitted as well. These results are displayed in the Table 3.

**Table 3:** Multivariate logistic regression analyses for the prediction of the appearance of an adverse reaction after BoNT injection.

Variables	OR	*p*-value	95% CI
GMFCS (IV–V *vs*. I–III)	2.74	0.016	1.20–6.26
Epilepsy	2.35	0.046	1.02–5.44

According to these results, children with GMFCS IV–V had almost a 3-fold increase in the possibility of presenting an adverse event, when all other patient characteristics were similar, and those with an epileptic disorder had more than a 2-fold increase in the possibility of presenting an adverse event as compared to those without epilepsy, when all other patient characteristics were similar. 

## 4. Discussion

This retrospective cohort review was based on data that were collected during clinical care according to a protocol that over 13 years was changed in some important ways due to emerging practice guidelines and literature reports. The majority of the patients we chose to treat according to existing guidelines were children and adolescents with bilateral spastic CP and belonged to GMFCS levels I, II, III. Since we have adopted the multilevel approach in BoNT-A treatment, it is not surprising that higher BoNT-A doses per kilo were injected to children with bilateral *vs*. unilateral involvement and to children more severely affected. BoNT-A doses per kilo were stable across sessions. 

Our practice has changed in several important ways according to emerging practice guidelines and literature reports during the 13 years of BoNT-A utilization by our group. The changes that are relevant to this report are: (a) the doses per kg per session and total maximal doses per session for both BoNT-A formulations that we utilized (Onabotulinumtoxin A and Abobotulinumtoxin A); (b) the method of conscious sedation. 

The first change resulted from the increased security we felt with higher Onabotulinumtoxin A doses while the opposite was true with Abobotulinumtoxin A. Our clinical observations were supported by emerging literature and as a result our practices were drastically changed with regards to Abobotulinumtoxin A doses per kg (20–30 IU/kg/session) and maximal total doses (up to 1500 IU/session). One of the problems we encountered from the outset was the appropriate conversion ratio of Onabotulinumtoxin A to Abobotulinumtoxin A and if a conversion rate in fact existed since the two preparations of BoNT-A are pharmacologically different. Other groups have discussed the need to adopt a different conversion ratio from the one they initially tried and this is presently believed to be closer to 1:1.7 (Onabotulinumtoxin A: Abobotulinumtoxin A) [21]. Our results on the doses of the two formulations for the muscles of the lower and upper limbs demonstrate that in practice we adopted a conversion rate closer to 1:2 and this was most likely the result of the maximum Abobotulinumtoxin A dosages that were adopted for safety purposes. Regarding the recommended dose levels of Onabotulinumtoxin A several reports have supported higher doses than the ones initially recommended by the company [17,18]. An international consensus statement published in 2010 concluded that the recommended doses are intermediate between previous consensus statements [7]. A number of studies have compared Onabotulinumtoxin A and Abobotulinumtoxin A. Several have been conducted in various types of dystonia in adults and have demonstrated similar efficacy of the two preparations, may be longer effect for Abobotulinumtoxin A, as well as, higher adverse event rate [22]. There are no analogous studies in cerebral palsy that we know of. Even though we had a rather limited number of adverse events even with high Abobotulinumtoxin A doses, our experience has led us to believe that Abobotulinumtoxin A, 20–30 IU/kg/session and maximal total doses (up to 1500 IU/session) are both efficacious and safe.

As for the second change regarding buccal midazolam usage for conscious sedation, we can comment positively on its ease of application and on its safety but we have not evaluated pain relief as such. There is certainly no established standard of care for sedation of patients undergoing botulinum toxin injections and great variability exists among spasticity clinics for pain prevention protocols, ranging from no intervention, to topical anesthesia, oral sedation, nitrous oxide or general anesthesia [23,24,25]. Both of the regimens we have employed appear safe with only five adverse events in 1382 treatment sessions, all in GMFCS V patients, once again emphasizing the need for extreme care in this very vulnerable group of patients. Parents at least in this country favor conscious sedation over general anaesthesia; yet, for some patients it would definitely be preferable to employ either deeper sedation or general anaesthesia for many reasons related to the patient (elimination of pain and anxiety) and to the treatment needs (better examination, iliopsoas injection, muscle localization by US technique). 

In this study, adverse events were reported in a small percentage of treated children and in a small percentage of performed injections. Most of them were attributed to the toxin, although at least five seemed to be associated with the drug selected to induce the pre-procedural sedation. This rate of adverse events is in accordance with previously published studies [26], even with those in which the injections were performed on the youngest patients, e.g., those younger than 2 years [27]. However, higher or lower rates have also been described [12,15,21,28,29]. In the study of Lowe *et al*., no adverse events attributed to BoNT-A injections were reported [29]. In accordance to the latter, A.lbavera-Hernandez *et al.* in their review, found five more studies reporting no adverse events from the toxin use [14]. 

All adverse effects reported in this study were also reported previously by other authors, with the exception of the occurrence of hypertonia that we believe was most likely the result of unopposed increased muscle tone in non injected muscles. The presence of some serious and even life threatening adverse reactions resulted in imperative efforts to discover possible factors responsible for that. The dosage scheme was first examined. In their study in 2001, Bakheit *et al.* found that total dose injected rather the dose calculated on the basis of body weight seem to be associated with adverse reactions. They have found that the highest incidence of adverse events was observed in patients receiving higher (>1000 IU) doses as compared to those injected with lower total doses (≤250 IU) [26]. More recently, Naidu *et al.* explored the incidence of systemic reactions in the treatment with BoNT-A. They also correlated increased doses of injections with the incidence of adverse reactions, especially bladder or bowel incontinence and unplanned hospital admissions for respiratory symptoms [12]. In contrast, in their study, Willis *et al.* found no increase in the adverse events incidence when doses 15–25 IU/kg were used in the lower limbs [30]. In monkeys, there were no observable systemic effects at doses below 33 IU/kg. However, there was toxicity progressing to death at doses of 38–42 IU/kg [31].

Recent guidelines had set the upper limit for BoNT-A to 25 IU/kg for both utilized regimens [18] although in multilevel treatment, it has been reported that doses up to 40 IU/kg (for Onabotulinumtoxin A) are safe [32]. In their consensus statement, Love *et al.* suggested use of doses that lie in an intermediate level between previous consensus statements [7]. It has been also recommended that total dosage should be limited to less than 18 IU/kg (for Onabotulinumtoxin A), in children with severe spastic quadriplegia who have dysphagia [33]. Finding the best upper dose limits for children at all GMFCS levels, particularly those at levels IV and V is still an important topic of research. Besides doses of BoNT-A used, it is suggested that the level of impairment may be associated to the presence of an adverse reaction [12]. Adair and Graham found that the incidence of adverse events increased sequentially from GMFCS I to GMFCS V [34]. Spreading of the toxin to remote areas of the body and/or the co-morbidities associated with more severely affected children may play a crucial role to that [11,12,13]. Based on these results, caution and careful monitoring is recommended when children severely affected, particularly those at levels IV and V with history of aspiration and respiratory disease, are treated with BoNT-A [7,12,14]. However, there is insufficient evidence to warrant restriction of the administration of BoNT-A in those children [13]. 

In this study, in accordance to the previous reports, we correlated the incidence of an adverse reaction with the severity of impairment. GMFCS IV-V levels were more frequently associated with the presence of an adverse reaction as compared to GMFCS I-III. However, doses used, either total or calculated per kilo, did not directly influence the possibility of presenting an adverse reaction. The quite small percentage of reported adverse reactions and the retrospective nature of this study may be the reasons for that. It should be noted though that in this study, children with GMFCS IV–V were injected with higher total doses per kilo as compared to GMFCS I–III. Nevertheless, total doses in more severely affected patients did not exceed maximum recommended doses (for Onabotulinumtoxin A: median doses 290 IU (200–380 IU, max: 600 IU and for Abobotulinumtoxin A: median doses 565 IU (360–840 IU, max: 1500 IU, results not shown).

An interesting finding though in our study, is that children with a history of epilepsy were more likely to present an adverse event after a BoNT-A injection. To our knowledge, this is the first time that this association is reported. Whether this correlation reflects the more severe impairment of children with CP and epilepsy or it is attributed to the concomitant antiepileptic medications those children need to receive, it is not clear. 

The major limitation of this study was that all adverse events except from those related to the sedation and observed during the patients’ short hospital stay, were reported and recorded during the 3-monthly follow-up visits. As a result, the accuracy of the reported events could be limited not to mention that the temporal relationship is difficult to assess retrospectively. Such problems are usually resolved in a prospective study design, where the follow-up is better organized and adverse events are closely observed. Another limitation is that in this type of intervention parents frequently report as an adverse event changes on muscle tone that are in reality the desired effect of the treatment. In addition, excessive weakness is not well defined and is rather subjective. 

## 5. Conclusions

Adverse events were rare; a few were related to the conscious sedation regimen. BoNT-A doses, either total or calculated per kilogram, did not correlate with adverse reactions. Increased incidence of adverse reactions was associated with GMFCS level and the presence of an epileptic disorder. These results indicate that treatment with BoNT-A was safe and that, when following established practice guidelines, adverse reactions both mild and relatively more serious did not produce major problems, even for the more severely affected patients. Education of patients and parents both for the expected results of the treatment and the potentially life-threatening adverse effects is of great value. From the physician's point of view, the knowledge of the general health status of the patient, comorbid conditions and concomitant medications is essential in identifying patients that have a higher probability of presenting an adverse reaction, especially a serious one.
